# Knowledge and practice of healthcare providers and associated factors of essential newborn care in Ethiopia: a systematic review and meta-analysis

**DOI:** 10.3389/fped.2024.1432582

**Published:** 2024-10-28

**Authors:** Temesgen Geta Hardido, Bizuayehu Atinafu Ataro, Eshetu Elfios, Tewodros Alemayehu Abuye, Christian Kebede

**Affiliations:** College of Medicine and Health Science, Wolaita Sodo University, Sodo, Ethiopia

**Keywords:** Ethiopia, healthcare providers, essential newborn care, systematic review and meta-analysis, practice, knowledge

## Abstract

**Background:**

Preventing neonatal mortality and morbidity in developing countries such as Ethiopia requires improved practices and knowledge among healthcare providers. Several studies have been conducted in Ethiopia, but the overall level has not been estimated based on essential newborn care practices, knowledge of health care providers, and associated factors. Therefore, the objective of this review is to assess the overall practice and knowledge of essential newborn care and associated factors among healthcare providers in Ethiopia.

**Methods and materials:**

Only articles published in English were included in this review. Medline/PubMed, Web of Science, Google Scholar, EMBASE, and CINAHL, Scopus, Ethiopian University Repository Online, and the Cochrane Library are the main databases. The review included cross-sectional studies written in English that met the inclusion requirements. Using a random-effects model, the overall practice and knowledge level was estimated. Additionally, funnel plots and Eggers’ test were used to assess publication bias. STATA version 14 was used to perform all statistical analysis.

**Results:**

This review included 15 studies involving 3,210 health care providers in Ethiopia. In Ethiopia, overall health care providers had a good practice and knowledge level of essential newborn care of 57.38% [95% CI (49.56; 65.20); *I*^2^ = 95.3%, *P* < 0.001] and 54.06% [95% CI (45.07; 63.05); *I*^2^ = 95.5%, *P* < 0.001], respectively. Knowledge, training status, and material availability of healthcare professional were significantly associated with their practice of essential newborn care, while educational qualification and training status were significantly associated with the knowledge of healthcare providers of essential newborn care.

**Conclusions:**

Overall, 57% and 54% of healthcare providers had good ENC practices and knowledge. So, the Ethiopian government and other stakeholders should take immediate measures to improve essential neonatal care practice and knowledge among healthcare providers, and improve identified factors.

## Introduction

Children are at highest risk of death in the first month of life. The newborn period is the most vulnerable period for children. Universal and high-quality newborn health care is the right of every newborn, everywhere. Newborns have the right to be protected from injury and infection, to breathe normally, to be warm and fed. All newborns should have access to essential newborn care, that is, essential care for all newborns in the first days after birth. Essential newborn care (ENC) includes immediate care at birth and essential care throughout the neonatal period. It is needed both in health care settings and at home (1, 2). To promote newborn health, the World Health Organization has recommended key components of ENC, such as immediate drying and supplemental stimulation, neonatal suction, cord clamping, skin-to-skin contact skin, starting breastfeeding, vitamin K prophylaxis, keeping the newborn warm, and eye care (3).

Ethiopia is one of the developing countries with the highest rates of neonatal mortality. According to recent Ethiopian data; four out of ten newborns die during the early neonatal period. Implementing one or more of the four essential elements of neonatal care is linked to greater neonatal survival. It also reported that the use of ENC could reduce infant mortality by 72% (4, 5). In many developing countries, physicians, nurses, and midwives play an important and diverse role in reducing maternal and newborn mortality by providing adequate antenatal care, labor and delivery, and ongoing newborn care. Health care providers, in particular, provide skilled support during the birth process, perform neonatal resuscitation, provide critical newborn care, and stabilize at-risk and sick newborns, including determining whether they need to be transferred to a nearby hospital (6).

Many sub-Saharan African countries face a shortage of essential newborn care supplies and equipment, exacerbated by a lack of knowledge and practice among health professionals (7). Health professionals’ knowledge and practice of health professionals of lifesaving newborn care was determined by their profession, qualification, interest in working in the delivery room, training on the job, year of training, and access to a national guideline in their institution (8, 9). Poor practice and knowledge of health care providers have a negative impact on providing an effective ENC, which significantly increases neonatal mortality and morbidity (10, 11). Nurses and midwives must have the necessary knowledge and skills to provide immediate newborn care intervention (12–14).

Despite the fact that most health professionals (93%) received in-service training on immediate newborn care, nearly half of them (50.25%) had inadequate knowledge (15). In a single study conducted in Sub-Saharan countries, 56% of doctors, 50% of mid-level morbidity caused by ineffective ENC provided by unskilled health professionals, as well as regularly assessing health providers’ knowledge and practice levels, as well as associated factors regarding ENC (16).

Previous literature has shown that the knowledge of healthcare professionals about ENC is 72% in Pune (17), 64% in Vietnam (18), 69.2% in Egypt (19), 74.65% in Tigray (20), 61.7% in Sidama (21), 56% in Amhara (22), and 53% in Pakistan (23). In terms of practice, the level of practice level was 59.7% in Amhara (22), 55% in India (24), 59.8% in Tigray (25), and 68.3% in Oromia (26). Factors such as working in a hospital, being a woman, interested in providing care to newborns, the level of education, interest in working in a delivery room, and the existence of guidelines were significantly associated with the knowledge of ENC among healthcare providers (10, 11, 21, 22, 26–28). On the other hand, work experience, inadequate knowledge, workload, not being interested in providing immediate newborn care and working in a health center, availability of national guidelines, adequate materials, access to training, knowledge of essential newborn care, type of facility, and experience working in a delivery unit were significant predictors of the practice of newborn care by healthcare providers (11, 20, 22, 26, 27)

The World Health Organization (WHO) plans to reduce infant mortality to less than 12 per 1,000 live births by 2030 (29). However, medical providers have poor knowledge and practice in prenatal and newborn care (9). In general, nurses, midwives and nursing assistants are usually positioned to care for newborns admitted to formal health facilities (30). Thus, the improvement of their knowledge and skills is a very important aspect of these health facilities (26). The morbidity and mortality rate of newborns is reduced by improving access to educational information and providing appropriate treatment for women and newborns (31). Therefore, proper care of newborns by qualified and knowledgeable healthcare providers is essential for survival, growth and development of newborns (32). However, newborn mortality is unacceptable at birth in Ethiopia and the state of newborn care practice and knowledge among healthcare providers is not well understood in Ethiopia in general (4, 33).

This is the first systematic review to address important knowledge gaps in the knowledge and practice of ENC among health care providers in Ethiopia. This study will help to be used as evidence to evaluate the Sustainable Development Goals to reduce neonatal mortality by improving the quality of essential newborn care in developing countries. Although several separate studies have been conducted to evaluate the general status of knowledge and practice of immediate care for new births among health care providers in Ethiopia, there were no national data supporting the general status of knowledge and practice and related factors associated with ENC among health care providers in Ethiopia (10, 11, 20–22, 25–28, 33–38).

The representativeness and results of a single study are neither conclusive nor consistent. Therefore, the purpose of this systematic review and meta-analysis was to assess the magnitude of knowledge and practice and the associated factors of essential newborn care among health providers in Ethiopia. The results of this study provide general information that helps reduce infant mortality in children by helping to inform policies, design strategies, and improve the ENC. This plays an important role in reducing neonatal mortality. The review was intended to answer the following research questions. (1) What is the level of knowledge and practice of health professionals in Ethiopia? (2) What are the associated factors that affect the knowledge and practice of health care providers in ENCs in Ethiopia?

## Methods and materials

### Study design and area

A systematic review and meta-analysis was conducted to estimate the overall level of the practice and knowledge of ENC and associated factors among health care providers in Ethiopia.

### Search strategy

Databases such as Medline/PubMed, Web of Science, Google Scholar, Scopus, EMBASE, and CINAHL, the Ethiopian University Repository online and the Cochrane Library were used to search for the studies from March 1 to 30, 2024. We checked the database at (http://www.library.ucsf.edu) and the Cochrane Library to ensure that this study was not conducted and to avoid duplication of efforts. PROSPERO also registered this review with the registration number of CRD42024544479. After confirming that a similar had not previously been conducted in Ethiopia, a comprehensive search strategy was developed using multiple Boolean operators on standard population exposure and outcome (PEO) questions. The words “or” and “and” were used to combine search terms. The terms “Essential newborn care” and immediate newborn care”, AND “practice”, AND “knowledge”, AND “associated factors” or “determinants” or “influencing” OR “health care providers” OR “health care professional” OR “midwives” OR “doctors” OR “nurse” AND Ethiopia are searched using Boolean operators. Those papers met the inclusion criteria in terms of title, and the abstracts were read in their entirety. Three authors (TG, CK, and TA) carried out the search strategy. All articles retrieved from the database are checked for titles and abstracts before being exported to the Endnotes library. These articles met the inclusion criteria in terms of titles and abstracts that were read in full. Three authors (TG, EE, and BA) performed a search strategy. We strictly follow the Preferred Reporting Items for Systematic Review and Meta-Analysis (PRISMA) protocol to estimate the general status of the knowledge and practice of health care providers about ENC and associated factors in Ethiopia.

### Operational definition

The operational definition of the outcome variable in the research is similar. Many papers stated that practice is good if the provider practices more than or equal to 70% of listed procedures\practice or poor if it is less than 70%. Likewise, they stated provider's knowledge as adequate or good if the providers answered questions related to knowledge above or equal to the mean score. And if their answers were lower than the average score, they would have poor or inadequate knowledge (10, 11, 20–22, 25–28, 33–38).

### Eligibility criteria

#### Inclusion and exclusion criteria

The articles eligible for this review were those that assessed the general level of knowledge and practice of healthcare providers and its associated factors of ENC in Ethiopia. In this review, the studies conducted in a cross-sectional study design and published in English were included. Additionally, it included participants who lived in Ethiopia. It also included studies conducted from 2014 to 2024. Studies that did not address the level of knowledge and practice of healthcare providers were excluded. This review excluded studies conducted outside Ethiopia and study designs other than cross-sectional studies.

### Data extraction

PRISMA was used to select and direct the selection of articles for this review. The parameters used to extract the data include the author's name, publication year, and study location, sample size for each study, study population, and study design and outcome. Using a Microsoft Excel spreadsheet, we collected the required data from the accepted articles. Three authors (TG, TA, and EE) independently extracted information from the supplemental documents. The studies that met the admission requirements were included after detailed agreement and discussion for data extraction and summarized in the table (Table 2).

### Assessment of risk of bias and quality

To assess the quality of the study, a critical analysis was performed using the Joanna Brings Institute Review Meta-analysis and Statistical Evaluation Tool. Joana identifies the studies and abstracts of the articles to decide whether they should be included. The quality of the articles was evaluated before selecting the final review. Cross-sectional studies were evaluated based on the source population, the adequacy of the sample size, data collection methods, data collection tools, statistical analysis, and the adequacy of the response rate, and scored on one to nine point scales. A score of a quality assessment indicator of seven or more was considered low risk for this review (Table 1).

**Table 1:** Critical appraisal results of eligible studies for the review on knowledge and practice towards ENCs among health care providers in Ethiopia, 2024 (*n* = 15).

Yosef et al. (10)	Q1	Q2	Q3	Q4	Q5	Q6	Q7	Q1	Q9	Total
Tasew et al. (25)	Y	Y	Y	Y	Y	Y	Y	Y	Y	9
Gebru et al. (37)	Y	Y	Y	N	Y	Y	Y	Y	Y	8
Berhe et al. (20)	Y	Y	N	Y	Y	Y	Y	Y	Y	8
Yemaneh et al. (22)	Y	Y	Y	Y	Y	Y	Y	Y	Y	9
Arba et al. (27)	Y	N	Y	Y	Y	Y	Y	Y	Y	8
Ayenew et al. (38)	Y	Y	Y	Y	Y	Y	Y	Y	Y	9
Sofiya et al. (35)	Y	Y	Y	Y	Y	N	Y	Y	Y	8
Negussie et al. (26)	Y	U	Y	Y	Y	N	Y	Y	Y	8
Chanie et al. (33)	Y	Y	Y	Y	Y	Y	Y	Y	Y	9
Gebrehana et al. (36)	Y	N	Y	Y	Y	Y	Y	Y	Y	8
Abdu et al. (11)	Y	Y	Y	Y	Y	Y	Y	Y	Y	9
Diriba et al. (34)	Y	Y	Y	Y	Y	N	Y	Y	Y	8
Dejene et al. (21)	Y	U	Y	Y	Y	N	Y	Y	Y	8
Arba et al. (28)	Y	Y	Y	Y	Y	Y	Y	Y	Y	9

Y, yes; N, no; U, unclear; JBI critical appraisal checklist for studies reporting prevalence data: Q1 = was the sample frame appropriate to address the target population? Q2-Were study participants sampled appropriately? Q3-Was the sample size adequate? Q4-Were the study subjects and the setting described in detail? Q5-Was the data analysis conducted with sufficient coverage of the identified sample. Q6-Were the valid methods used for the identification of the condition? Q7-Was the condition measured in a standard, reliable way for all participants? Q8-Was there appropriate statistical analysis? Q9-Was the response rate adequate, and if not, was the low response rate managed appropriately?

### Data processing and analysis

A Microsoft Excel spreadsheet was used to extract the data, and STATA version 14 was used to analyze the data. Using random-effects model analysis, the pooled level of knowledge and practice of ENC among healthcare providers in Ethiopia was calculated. With the aid of a funnel plot and visual analysis, publishing bias was investigated. The heterogeneity of the study was tested using Cochrane Q-Static and I2. The general status of the knowledge and practice of ENC by healthcare providers in regions was compared with an estimated prevalence using a subgroup analysis. A Forest pilot with 95% CI was used to show a pooled status. Meta-analysis was done to assess association between factors and the level of the knowledge and practice of ENC. *P*-values less than 0.005 was considered as a significance level.

## Results

### Identification and characteristics of included studies

**Figure 1:**
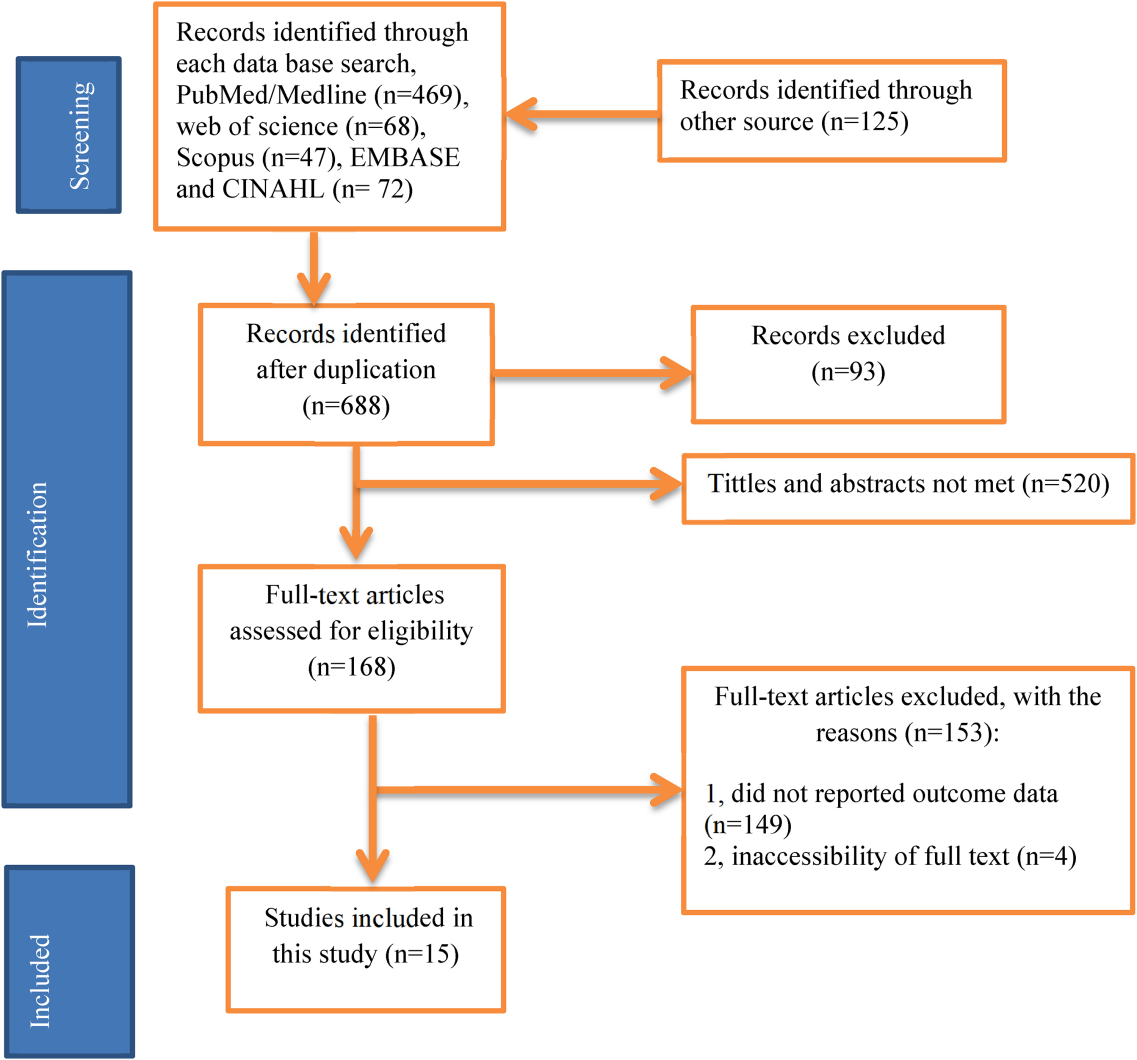
PRISMA flow diagram of study selection for systematic review of health professionals’ practice and knowledge on ENC in Ethiopia, 2024(*n* = 15).

From1 to 30 March 2024, 781 articles were identified in major electronic databases and other applicable sources. Of these identified items, 93 articles were eliminated due to duplication and 688 items were retained for further consideration. 520 studies were excluded because the abstract and title did not meet the requirements. Of the remaining 168 articles, 153 studies were excluded due to inconsistencies with the inclusion criteria established for that study. Finally, 15 studies that met the eligibility criteria were included in this study (Figure 1).

A total of 15 articles with 3, 210 participants were included in this systematic review and meta-analysis. For the practice of health professionals, the sample size was between 134 (22) and 272 (26). The sample size for the knowledge of healthcare professionals was ranged 134 (22) and 267 (21). Regarding the regional distribution of the included studies 3 in Tigray (20, 25, 37), 4 in the Amhara region (22, 33, 36, 38), 5 in the Southern Nations, Nationalities and Peoples Region (SNNPR) (10, 21, 27, 28, 35), one in Afar (11), Oromia (26), and Benshangul Gumuz (34) (Table 2).

**Table 2:** Study characteristics included in the systematic review of health care providers’ practice and knowledge towards ENCs in Ethiopia, 2024 (*n* = 15).

Author	Year	Region	Study area	Study design	Sample	Proportion	
						Practice	Knowledge
Yosef et al. (10)	2021	SNNPR	Benchimaji Kafa	Cross-sectional	157	61.8	38.2
Tasew et al. (25)	2019	Tigray	Northeast	Cross-sectional	179	64.8	59.8
Gebru et al. (37)	2019	Tigray	Centeral zone	Cross-sectional	147	52.4	17.7
Berhe et al. (20)	2017	Tigray	Eastern Zone	Cross-sectional	215	72.77	74.6
Yemaneh et al. (22)	2017	Amhara	Bahirdar	Cross-sectional	134	59.7	56
Arba et al. (27)	2020	SNNPR	Wolaita	Cross-sectional	216	44.4	-
Ayenew et al. (38)	2020	Amhara	Awi zone	Cross-sectional	201	62.7	-
Sofiya et al. (35)	2022	SNNPR	Arbaminch	Cross-sectional	195	24.1	59.5
Negussie et al. (26)	2018	Oromia	Jimma	Cross-sectional	272	51.1	52.2
Chanie et al. (33)	2021	Amhara	Gondar	Cross-sectional	214	74.8	-
Gebrehana et al. (36)	2021	Amhara	Northshoa	Cross-sectional	256	73.8	62.9
Abdu et al. (11)	2019	Afar	Afar	Cross-sectional	357	62.7	53.8
Diriba et al. (34)	2022	Benshangul Gumuz	Assossa	Cross-sectional	267	41.5	61.7
Dejene et al. (21)	2022	SNNPR	Sidama	Cross-sectional	182	56.6	-
Arba et al. (28)	2020	SNNPR	Wolaita	Cross-sectional	218	-	57.9

### The level of knowledge and practice of ENC among health care providers in Ethiopia

**Figure 2:**
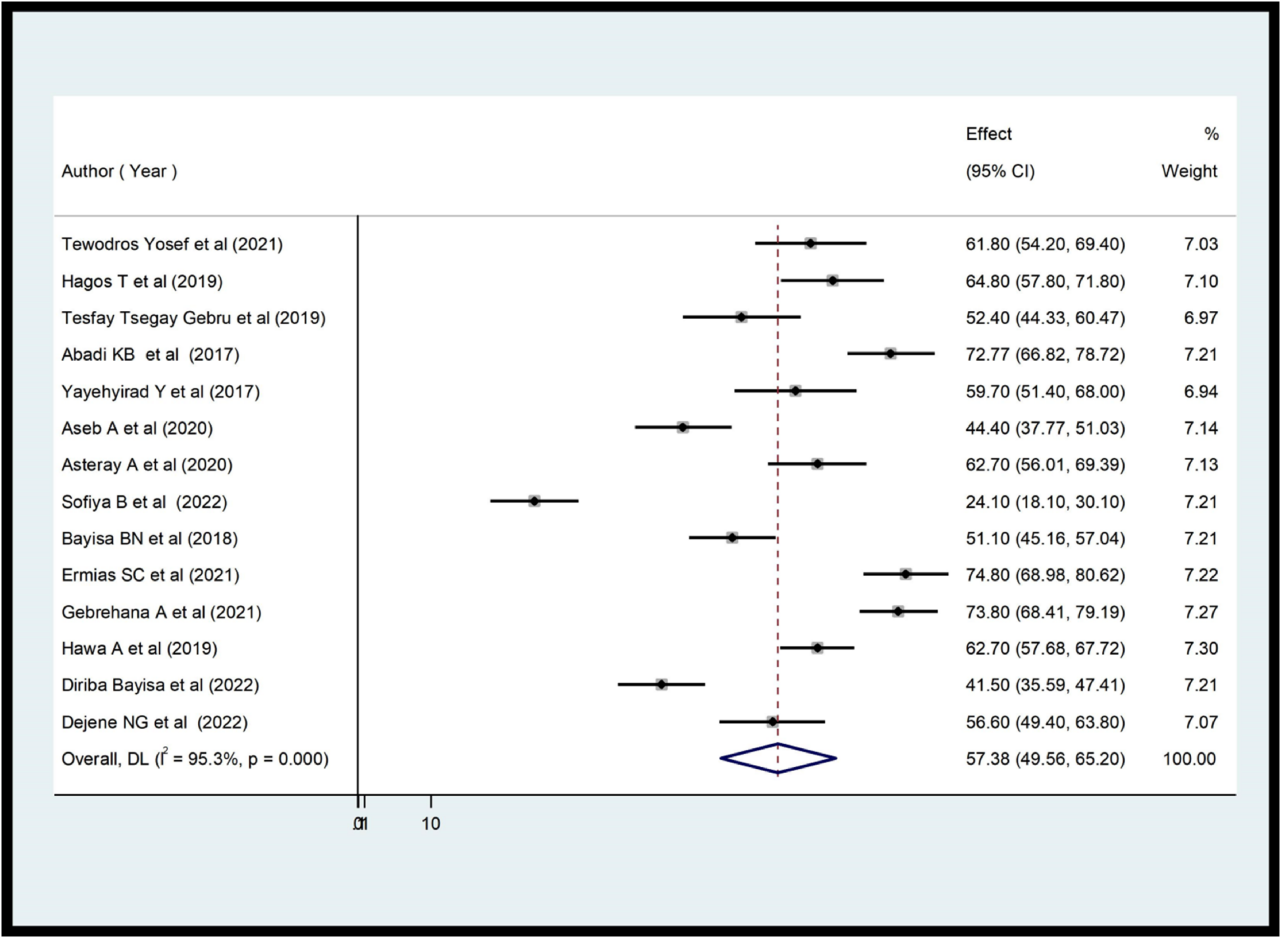
Forest plot showing the pooled prevalence of health care providers’ practice on ENC in Ethiopia (*n* = 14).

**Figure 3:**
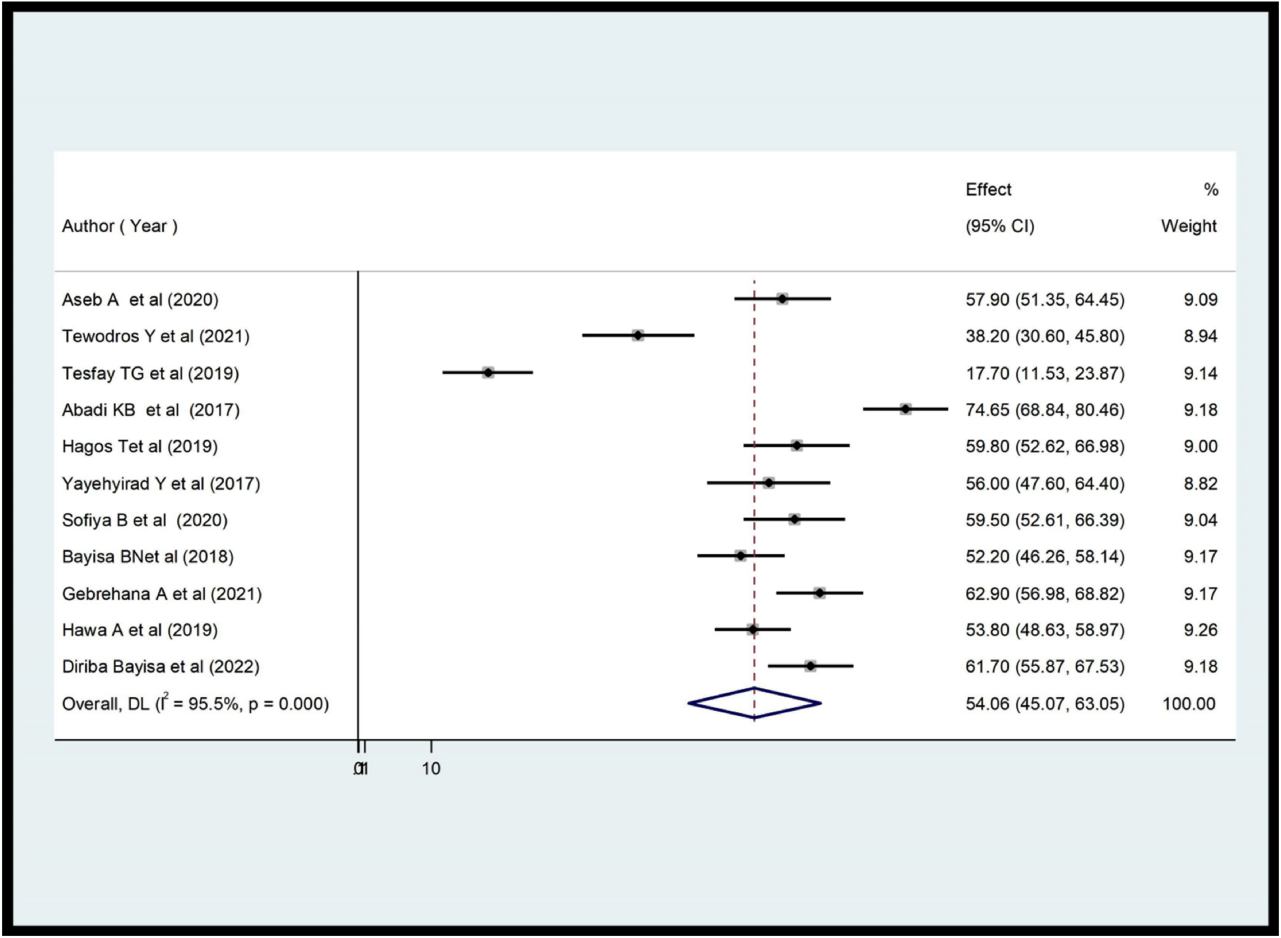
Forest plot showing the pooled prevalence of health care providers’ knowledge on ENC in Ethiopia (*n* = 11).

According to this study, the level of the practice of health care providers toward ENCs in Ethiopia ranged from 24.10% (35) to 74.8% (33). The overall prevalence of practice in Ethiopia was 57.38% [95% CI (49.56; 65.20); *I*^2^ = 95.3%, *P* < 0.001] (Figure 2). And also the level of knowledge of healthcare professionals in ENCs in Ethiopia was between 17.7% (37) and 74.65% (20). The overall level of knowledge of ENC among health care providers in Ethiopia was 54.06% (95.07; 63.05; *I*^2^ = 95.5%, *P* < 0.001) (Figure 3).

### Subgroup analysis of the practice and knowledge of health care providers about ENCs in Ethiopia

**Figure 4:**
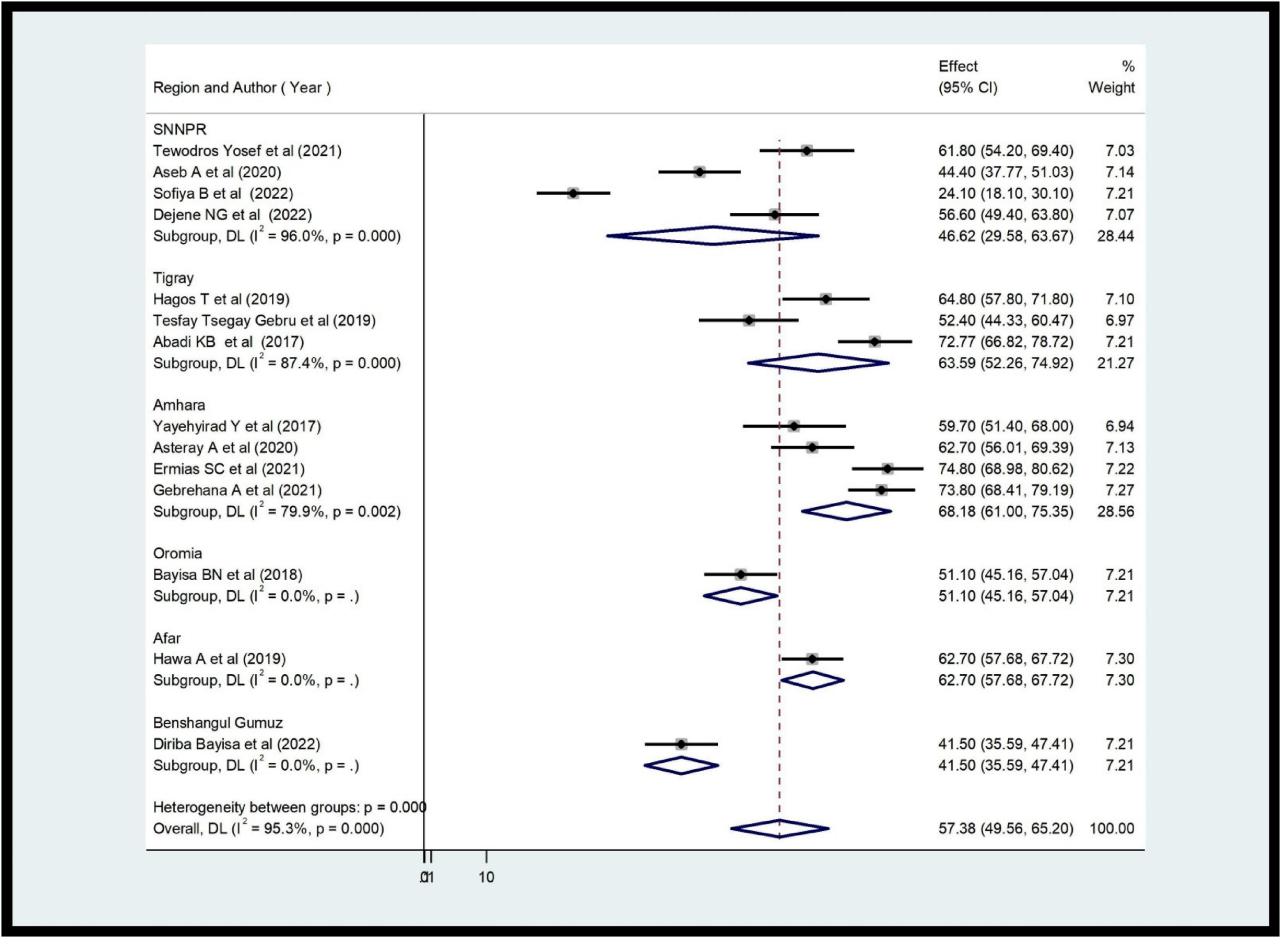
Subgroup analysis of providers’ practice about ENCs in Ethiopia (*n* = 14).

**Figure 5:**
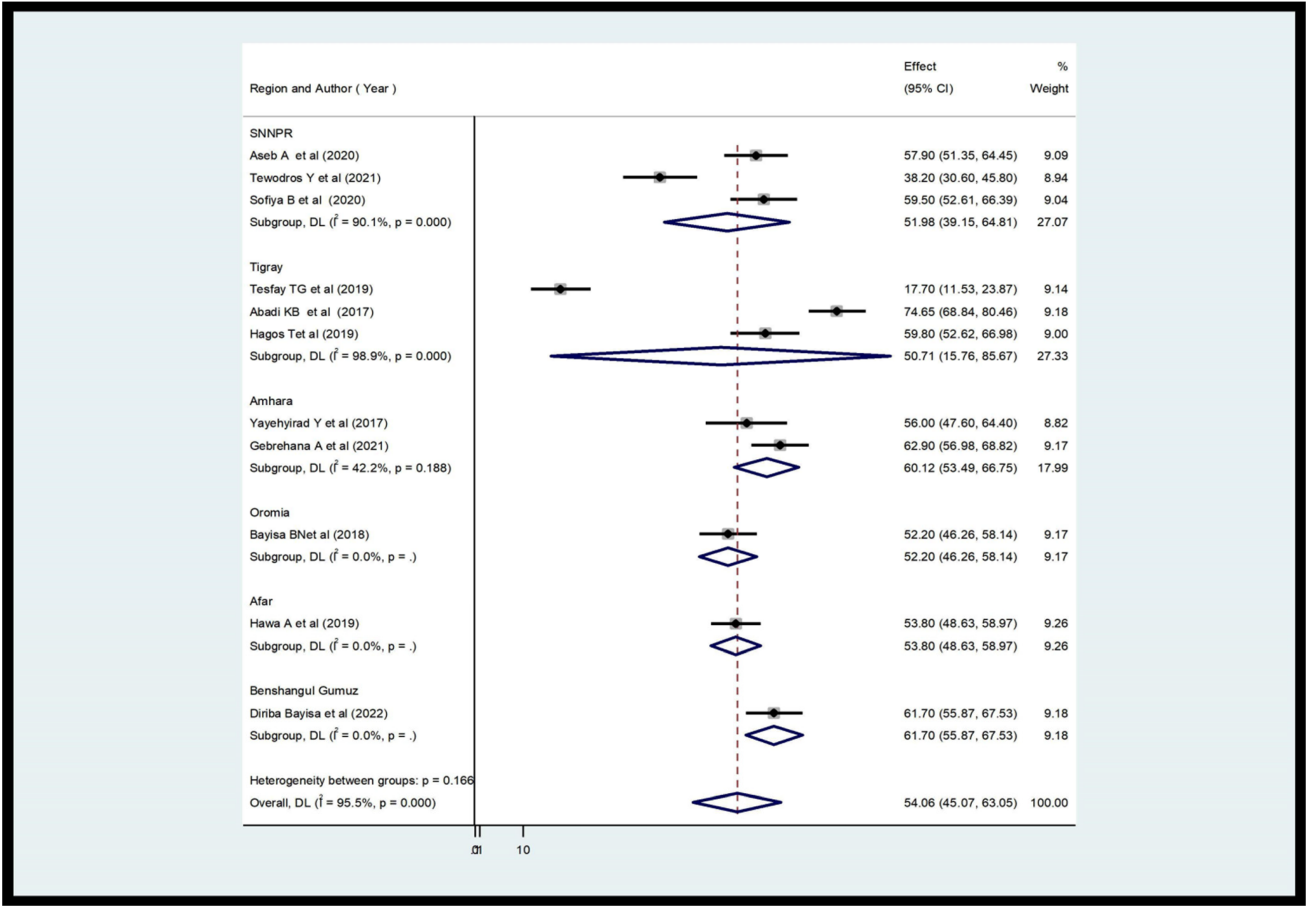
Subgroup analysis of the review on providers’ knowledge of ENCs in each region (*n* = 11).

After confirming the study heterogeneity, a subgroup analysis of the ENC practice of healthcare providers in each region revealed that Amhara had the highest level of provider practice with a score of 68.18% (95% CI 61.00, 75.35), followed by Tigray with 63.90% (95% CI 52.26, 74.94). The Benshangul Gumuz region had the lowest value, at 41.50% (95% CI 39.59, 47.11) (Figure 4). The subgroup analysis of health care providers’ knowledge revealed that Benshangul Gumuz had the highest level of ENCs with a score of 61.70% (95% CI 55.87, 67.55), followed by Amhara with 60.12% (95% CI 53.49, 66.75). The Tigray region had the lowest value at 50.71% (95% CI 15.76–85.67) (Figure 5).

### Heterogeneity and publication bias

**Figure 6:**
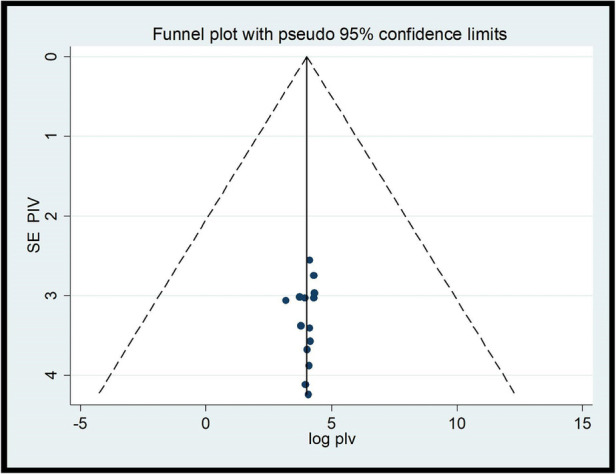
Funnel plot of the studies included in review on the providers’ practice about ENCs.

**Figure 7:**
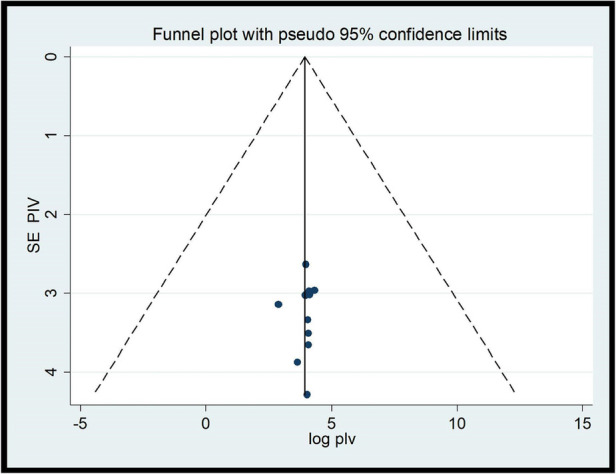
Funnel plot of studies included in the review on health care providers’ knowledge on ENCs in Ethiopia (*n* = 11).

To minimize and control the heterogeneity of the study, we conducted subgroup analyzes by region. The results of the I2 test show that there was significant heterogeneity between the studies (*I*^2^ = 99.3%, *P* < 0.001). The publication bias of studies on the providers’ ENC practices was checked with the Eggers test and visual inspection of a funnel plot. The results of the funnel diagram showed that the selected studies had a symmetric distribution after inspection (Figure 6) and the Eggers test (*P* = 0.824). Both showed that there was no bias in the publication. Regarding studies, including studies on ENC provider knowledge, the results of the *I*^2^ test show that there was a significant heterogeneity between studies (*I*^2^ = 99.3%, *P* < 0.001). The publication bias of the studies was monitored with the Eggers test and visual inspection of a funnel plot. The results of the funnel plot showed that the selected studies had a symmetric distribution after inspection (Figure 7) and the Eggers test (*P* = 0.796). This showed that there was no publication bias.

### Factors associated with the practice and knowledge of health care providers of ENCs in Ethiopia

In this review, three variables (Knowledge, training status, and material availability) were significantly associated with the practice of providers towards ENC, while supervision, providers’ interest in providing ENC, educational qualification, and providers’ experience were not significantly associated with the practice of ENC. Educational qualification and training status have a statistical association with provider knowledge, whereas type of health facility, providers interest in providing ENC, and material availability have no effect on provider knowledge of ENC.

The results of the analysis showed that there was a significant association between the practice of ENC healthcare providers and the knowledge of health professionals. Health professionals with good knowledge were almost two times more likely to practice ENC than those with poor knowledge [OR = 1.95, CI (1.11, 3.43), *I*^2^ = 81.7%, *p* = 0.000]. This review also showed that there was a significant association between provider training and the practice of ENC. The practices of healthcare providers were almost 19 times higher among those who had been trained than among those who had not been trained [OR = 18.87, CI (2.02,176.1), *I*^2^ = 98.2%, *p* = 0.000]. Furthermore, providers who had adequate ENC materials were 3.35 times more likely to practice ENC than providers who did not [OR = 3.35, CI (1.93, 4.77), *I*^2^ = 77.8%, *p* = 0.004]. Regarding the knowledge of the ENC providers, providers with a B.Sc. and higher education levels were 26.58 times more likely [OR = 26.58, CI (1.32, 55.0), *I*^2^ = 81.5%, *p* = 0.003] to have sufficient knowledge of ENC than their contrast (Table 3).

**Table 3:** Factors associated with health care providers’ practice and knowledge towards ENCs in Ethiopia, 2024 (*n* = 15).

Significant and non-significant factors	OR	CI	*I* ^2^	*P*	Outcome
Health professionals’ knowledge (Good)	1.95	(1.11, 3.43)	81.7%	**0**.**000**	Practice
Training (Yes)	18.87	(2.02, 36.1)	98.2%	**0**.**000**	Practice
Supervision (Yes)	8.82	(1.92, 39.0)	81.1%	0.450	Practice
Material availability (Yes)	3.35	(1.93, 4.77)	77.8%	**0**.**004**	Practice
Providers’ interest to provide ENCs (Yes)	2.81	(1.35, 4.28)	89.1%	0.741	Practice
Educational qualification (BSc and above)	1.53	(0.99, 2.37)	48.6%	0.120	Practice
Providers’ experience (>2 years)	0.44	(0.35, 0.54)	52.24%	0.124	Practice
Educational level (BSc and above)	26.58	(1.32, 55.0)	81.5%	**0**.**003**	Knowledge
Type of health facilities (Hospital)	101.49	(0.58, 178.41)	98.1%	0.080	Knowledge
Training (Yes)	2.00	(1.23, 3.48)	94.4%	**0**.**001**	Knowledge
Providers’ interest to provide ENCs (Yes)	16.67	(3.86,71.9)	85.9%	0.741	Knowledge
Material availability (Yes)	9.67	(5.27, 17.73)	82.6%	0.99	Knowledge

OR, odd ratio; CI, confidence Interval; *I*^2^, Chi-square; P, *p*-value.

Bolded *p*-value, significant level.

Finally, trained providers were 2.5 times more likely to have adequate knowledge of ENC than the untrained group [OR = 2.50, CI (1.23, 3.48), *I*^2^ = 81.5%, *p* = 0.003] (Table 3).

### Review outcomes

The first outcome from this systematic review and meta-analysis is an estimate of the overall level of ENC practice and knowledge in Ethiopia. The general level of ENC practice and knowledge in Ethiopia was estimated, and 57% and 54% of Ethiopian providers were found to have good ENC practice and knowledge, respectively. The second outcome concerns factors related to ENC practice and knowledge among providers (Table 3). Results showed that healthcare professionals’ knowledge, equipment availability, and provider education and training were all predictors of ENC practice and knowledge.

## Discussion

Newborns receiving the standard ENC decreased global average deaths per 1,000 live births from 37 in 1990 to 17 in 2022. Despite this improvement, the mortality rate of newborns has remained high in all countries. The mortality rate for children born in sub-Saharan Africa is 10 times higher in the first month than for children born in high-income countries. A good health care providers practice ad knowledge level could have a significant effect to reduce neonate mortality and morbidity (2).

According to a systematic review and meta-analysis, 57.38% of Ethiopian providers demonstrated good ENC practice [95% CI (49.56; 65.20); *I*^2^ = 95.3%, *P* < 0.001]. This study is supported by previous studies done in Sidama (56.6%) (21), Bahirdar (56%) (22), and Nigeria 62.9% (39). However, this result is higher than previous studies conducted in Benshangul Gumuz (41.5%) (34), Arbaminch (24.1%) (35), Wolaita (44.4%) (27), and lower than the studies conducted in Pune city 98% (40), 74.8% in Gondar (33), 74.65% in Tigray (20), Northeast Amhara (73.8%) (40). The review found that Ethiopian healthcare providers had an estimated pooled knowledge of ENC of 54.06% [95% CI (45.07; 63.05), *I*^2^ = 95.5%, *P* < 0.001]. This review was consistent with previous studies (11, 21, 22, 37). However, studies conducted in Tigray (74.65%) (20), Amhara (62.9%) (40), Northeast Amhara (74.68%) (20), and Benshangul Gumuz (61.7%) (34) yielded higher results than the current review. In contrast, this review was higher than previous studies conducted in various parts of Ethiopia, including Tigray (17.7%) (37) and southern Ethiopia (38.2%) (10). The discrepancy occurred for both practice ad knowledge level could be explained by differences in the infrastructure of health facilities, the type of healthcare provider, the training status, the study period, the sample size, the methodology used in the study and the educational level and experience of the healthcare providers.

The review also provided a report on the knowledge and practices of the ENC of health providers throughout the region. A subgroup analysis showed that 68.18% of professionals practiced in Amhara, while a low percentage (41.5%) was observed in the Benshangul Gumuz region.

Possible reasons for these discrepancies include the experience and training of health providers, the availability and availability of ENC guidelines, supervision, and the quality of the high management system. The Amhara region is one of Ethiopia's developed regions compared to Benshangul Gumuz. The region provides good health services compared to the Benshangul Gumuz region. Furthermore, the proportional differences in the included studies may influence the final results of the review. The research included from the Amhara region has the highest level of practice compared to Benshangul Gumuz. This helps improve the practice of ENC providers. When it comes to ENC knowledge across the region, Benshangul Gumuz had the highest score (61.70%), while the Tigray region had the lowest (50.7%). The attitude and perception of the providers towards acquiring new knowledge, the prevalence difference of the studies included could lead to this discrepancy between regions.

According to this study, there is a similar level of practice and knowledge in Ethiopia. This may be because providers with good knowledge are more likely to practice ENC. Therefore, health care professionals with sufficient knowledge can strengthen confidence in performing their duties. Likewise, those who have good practices can have adequate knowledge. Because before providing healthcare services, caregivers must have good knowledge.

Knowledge, training status, and material availability of healthcare professionals were identified as important factors influencing their practice in ENC. Health professionals with good knowledge were about twice more likely to practice ENC than those with poor knowledge. Previous research has supported this finding (26, 27, 35). The possible reason is that the knowledge of health care providers is required to provide effective health care services (40). Furthermore, health care providers with adequate knowledge were more active and dedicated to effectively practicing ENC (27). As educational status of healthcare providers improves, their knowledge-enhancing behavior about ENC will also increase. As a result, they are better prepared to understand the health status of the newborn and risk factors for illness. Furthermore, more educated healthcare providers may have better access to or be more persuaded to obtain evidence-based information about newborn well-being and develop better newborn care management strategies. Furthermore, having adequate knowledge of ENC makes it easier to understand the newborn complications (1, 3). This implies that to provide effective ENC, all health care providers must have sufficient knowledge.

There is a significant association between the practice of health care providers and their level of training. Those who received ENC training were 19 times more likely to practice it effectively. This review is consistent with previous research (21, 28, 33, 35). This could be due to the fact that training provides an excellent opportunity to gain new knowledge or update information. Furthermore, training can help healthcare professionals improve their skills and performance. Training will improve caregiver abilities, resulting in higher survival rates and better newborn care. As a result, any healthcare care provider who works in ENC rooms should be trained in ENC. When they trained, they practiced more. This is a good approach to preventing unexpected neonatal morbidity and mortality in low-income countries such as Ethiopia.

The last, but not least, predictor, significantly associated with the practice of healthcare providers, is material availability. Health care providers who had sufficient ENC materials were more likely to practice ENC than those who did not. Previous studies have been consistent with this review (33, 36). The availability of appropriate health care materials is one of the main indicators of the quality of health care services. If there are no or a few materials in the ENC room, this could lead to unnecessary referrals to other health facilities at the same level and lead to poor health care practices. The provision of sufficient materials and frequent supervision by the regional or federal health office is the key to improving the practice of health professionals in the ENC (33, 34). Furthermore, if there are limited materials (guidelines, drugs, and vaccines) in the health facilities, health care providers cannot obtain sufficient knowledge about essential newborn care. The hospital management should comply with standard materials such as guidelines, medications, and vaccines in the delivery and neonatal units of all staff to increase the level of practice of health care providers.

This analysis identified two factors, such as educational qualifications and training as important predictors of the knowledge of health care providers about the ENC. Those with a B.Sc. and above the educational level are more likely to have good knowledge about ENC than their contrasts. This result is comparable to previous studies (27). The possible explanation could be that the high educational level of healthcare providers could generally have greater decision-making power and competence in the implementation of essential newborn care. In addition, higher education of health providers could have the opportunity to acquire different types of training and skills that bring good practices in the essential care of newborns (33).

Finally, this review found a strong relationship between training and ENC knowledge. Care providers who received ENC training were more likely to have adequate knowledge than their counterparts. On-the-job training increased the knowledge of health professionals about newborn care by three times compared to those who did not receive it. Similar studies conducted in Ethiopia have supported this finding (20, 26, 41). This could be because on-the-job training allows health professionals to update their previous knowledge of essential newborn care.

Despite its multilevel importance, the current systematic review and meta-analysis were not without limitations. First, due to the cross-sectional nature of all included primary studies, the outcome variable can be influenced by confounders. Second, studies with small sample sizes may have a negative impact on the national estimate of ENC knowledge and practice among healthcare providers. Finally, only six regional states were involved due to the limited studies.

## Conclusions

Overall, 57% of healthcare professionals had good ENC practices and 54% had good ENC knowledge. In this review, three variables: Knowledge, training status, and material availability were significantly associated with providers’ practice toward ENC, while educational qualification and training status were significantly associated with health care providers’ knowledge of healthcare providers about ENC. As a result, Ethiopian governments, ministries of health, and non-governmental organizations were strongly encouraged to improve ENC by providing training, providing necessary materials, and upgrading the knowledge of healthcare providers about ENC.

## References

[1] World Health Organization. Neonatal Health; Essential Newborn Care (2017). Available online at: https://www.who.int/teams/maternal-newborn-child-adolescent-health-and-ageing/newborn-health/essential-newborn-care (accessed January 21, 2024).

[2] UNICEF Data: Monitoring the situation of children and women. Neonatal Mortality (2024). Available online at: https://data.unicef.org/topic/child-survival/neonatal-mortality (accessed January 21, 2024).

[3] World Health Organization. WHO Recommendations on Newborn Health Guidelines Approved by the WHO Guidelines Review Committee. Geneva: World Health Organization (2017). (WHO/MCA/17.07). License: CC BY-NC-SA 3.0 IGO. Available online at: https://iris.who.int/bitstream/handle/10665/259269/WH (accessed January 21, 2024).

[4] Bryce E Mullany LC Khatry SK Tielsch JM. Coverage of the WHO’s four essential elements of newborn care and their association with neonatal survival in Southern Nepal. BMC Pregnancy Childbirth. (2020) 20:540. 10.1186/s12884-020-03239-6 32938433 PMC7493414

[5] Tamir TT Asmamaw DB Negash WD Belachew TB Fentie EA Kidie AA Prevalence and determinants of early neonatal mortality in Ethiopia: findings from the Ethiopian demographic and health survey 2016. BMJ Paediatrics Open. (2023) 7:e001897. 10.1136/bmjpo-2023-001897 37208032 PMC10201239

[6] WHO. Newborn Nursing for Facility-Base Care Level II Units. Participatory Module-Based Learning Directed for Skill Upgrade. 3rd ed New Delhi: Division of Neonatology, Department of Pediatr, All India Institute of Medical Sciences (2014).

[7] De Graft-johnson J Vesel L Rosen HE Rawlins B Abwao S Mazia G Cross-sectional observational assessment of quality of newborn care immediately after birth in health facilities across six sub-Saharan African countries. BMJ Open. (2017) 7(3):e014680. 10.1136/bmjopen-2016-014680 28348194 PMC5372100

[8] Shridhar G Pandey A Karmani S. Evaluation of a multimodal teaching method on essential newborn care among health providers at a tertiary care hospital. Med J Armed Forces India. (2019) 75(3):303–7. 10.1016/j.mjafi.2018.06.004 31388234 PMC6676327

[9] Ayiasi RM Criel B Orach CG Nabiwemba E Kolsteren P. Primary healthcare worker knowledge related to prenatal and immediate newborn care: a cross sectional study in Masindi, Uganda. BMC Heal Serv Res. (2014) 14:1–11. 10.1186/1472-6963-14-65 PMC3931348 24511880

[10] Yosef T Getachew D Weldekidan F. Health professionals’ knowledge and practice of essential newborn care at public health facilities in Bench-Sheko zone, southwest Ethiopia. Heliyon. (2021) 7(11):e08369. 10.1016/j.heliyon.2021.e08369 PMC8608856 34849418

[11] Abdu H Gebrselassie M Abdu M Mare KU. Knowledge and practice of immediate newborn care among midwives and nurses in public health facilities of afar regional state, northeast Ethiopia. BMC Pregnancy Childbirth. (2019) 19:422. 10.1186/s12884-019-2581-3. 31744464 PMC6862785

[12] Scott JR Ronald SG Beth YK Arthur FH. Chapter 41. Danforth’s Obstetrics and Gynecology. 9th ed Baltimore: Lippincott Williams & Wilkins (2003).

[13] Cunningham FG Leveno KJ Bloom SL Hauth JC Gilstrap III LC Wenstrom KD. Williams Obstetrics. 22nd ed. New York: McGraw-Hill (2007).

[14] You D Lucia Hug Simon Ejdemyr Priscila Idele Daniel Hogan Colin Mathers Global, regional, and national levels and trends in under-5 mortality between 1990 and 2015, with scenario-based projections to 2030: a systematic analysis by the UN inter-agency group for child mortality estimation. Lancet. (2015) 386(10010):2275–86. 10.1016/S0140-6736(15)00120-8. 26361942

[15] NasorTaha AF. Assessment of knowledge, attitude and practices of nurse midwives towards immediate care of the newborn in Khartoum state teaching hospitals. J Am Sci. (2013) 9(9):263–270. Available online at: http://www.jofamericanscience.org (accessed January 21, 2024).

[16] Ameh CA Kerr R Madaj B Mdegela M Kana T Jones S Knowledge and skills of healthcare providers in sub-Saharan Africa and Asia before and after competency-based training in emergency obstetric and early newborn care. PLoS One. (2016) 11(12):e0167270. 10.1371/journal.pone.0167270 PMC5179026 28005984

[17] Devi YN Mahadalkar P Varghese R. To assess the knowledge and selfreported practices regarding immediate newborn care among the staff nurses from birthing units of selected hospitals of Pune city. Int J Appl Res. (2017) 3(6):499–502. Available online at: https://www.allresearchjournal.com (accessed January 21, 2024).

[18] Eriksson L Nga NT Målqvist M Persson L-Å Ewald U Wallin L. Evidence-based practice in neonatal health: knowledge among primary health care staff in northern Vietnam. Hum Resour Health. (2009) 7(1):36. 10.1186/1478-4491-7-36 19393073 PMC2678076

[19] Abd El Fattah N Negawa A El Dein Z. Assessment of quality of nursing care provided immediately after birth at university hospital. Life Sci J. (2009) 9(4):2115–26. Available online at: http://www.lifesciencesite.com (accessed January 21, 2024).

[20] Berhe AK Tinsae F Gebreegziabher G. Knowledge and practice of immediate newborn care among health care providers in eastern zone public health facilities, Tigray, Ethiopia, 2016. BMC Pediatr. (2017) 17(1):157. 10.1186/s12887-017-0915-8 28693501 PMC5504861

[21] Dejene NG Aberash ED Andargachew K Gemechu GB Amare TY Berhane EM Essential newborn care practice and associated factors among obstetric care providers of public hospitals in Sidama regional state, Ethiopia. SAGE Open Med:10:1–8. 10.1177/205031212210858 PMC8972914 35371485

[22] Yemaneh Y Dagnachew E. Knowledge and practice of immediate new born care (inc.) among health professionals in governmental health facilities of Bahir Dar city, north Ethiopia 2016. Qual Prim Care. (2017) 25(6):360–7.

[23] Begum R Riaz S Munir A Ghaffar T Bibi S. Assessment of knowledge of nurses and midwives regarding immediate newborn care. J Nurs Midwifery Sci. (2022) 2(01):6–10. 10.54393/nrs.v2i01.24

[24] Agrawal PK Agrawal S Ahmed S Darmstadt GL Williams EK Rosen HE Effect of knowledge of community health workers on essential newborn health care: a study from rural India. Health Policy Plan. (2012) 27(2):115–26. 10.1093/heapol/czr018 21385799 PMC3606030

[25] Tasew H Teshale T Bahrey D Mariye T Teklay G. Immediate newborn care of knowledge, practice and associated factors among health care providers in northwestern zonal health facilities Tigray, Ethiopia, 2018. BMC Res Notes. (2019) 12(1):427. 10.1186/s13104-019-4465-z 31315651 PMC6637593

[26] Negussie BB Hailu FB Megenta AD. Knowledge and practice of essential newborn care and associated factors among nurses and midwives working at health centers in Jimma zone, Ethiopia, 2016. J Nursing Care. (2018) 7(1):446. 10.4172/2167-1168.1000446

[27] Arba A Zana Z Alemayehu A Aschalew Z. Determinants of essential newborn care practice among nurses and midwives working at public health facilities in Wolayta zone, southern Ethiopia: observational study. Int J Pediatr Neonat Care. (2020) 6:168. 10.15344/2455-2364/2020/168 PMC7152963 32308691

[28] Arba A Zana Z. Knowledge of essential newborn care and associated factors among nurses and midwives: a cross-sectional study at public health facilities in Wolaita zone, southern Ethiopia, 2019. Int J Pediatr. (2020) 2020:3647309. 10.1155/2020/3647309 PMC7152963 32308691

[29] Liu L Oza S Hogan D Perin J Rudan I Lawn JE Global, regional, and national causes of child mortality in 2000–13, with projections to inform post-2015 priorities: an updated systematic analysis. Lancet. (2015) 385(9966):430–40. 10.1016/S0140-6736(14)61698-6 25280870

[30] Rutebemberwa E Pariyo G Peterson S Tomson G Kallander K. Utilization of public or private health care providers by febrile children after user fee removal in Uganda. Malaria J. (2009) 8(1):45. 10.1186/1475-2875-8-45 PMC2657913 19284673

[31] Bhutta ZA Ali S Cousens S Ali TM Haider BA Rizvi A Interventions to address maternal, newborn, and child survival: what difference can integrated primary health care strategies make? Lancet. (2008) 372(9642):972–89. 10.1016/S0140-6736(08)61407-5 18790320

[32] Gurung G. Practices on immediate care of the newborn in the communities of Kailali district. Nepal Med Coll J. (2008) 10(41–44). 18700631

[33] Chanie ES Kassaw A Senbeta M GebreEyesus FA. The shadow challenges to improve the state essential newborn care practices in healthcare providers: evidence from a multicenter cross-sectional study in Godar, Ethiopia. *BMC Pediatr.* (2021) 21(1):439. 10.1186/s12887-021-02903-w PMC8495988 34620140

[34] Diriba B Berhane T Mabratu D Hunduma D. Assessment of factors associated with practice and knowledge of essential newborn care among nurse and midwives in Assosa zone governmental health facilities, western Ethiopia, 2021. *Res Sq*. (2022). 10.21203/rs.3.rs-1210942/v1

[35] Sofiya B Godana W Miskir D. Immediate newborn care practice and associated factors among health care providers in Arbaminch town govermental health institutions of southern Ethiopia; facility based cross-sectional study. Pediatr Ther. (2022) 1. 10.35841/2161-0665.22.12.460

[36] Gebrehana A Akine E ,Betregiorgis Z Tadesse TT Mitku MT. Essential newborn care practice and associated factors among health care providers in Northeast Ethiopia: a cross-sectional study. Arch Public Health. (2021) 79:90. 10.1186/s13690-021-00613-4 34074312 PMC8167947

[37] Gebru TT Murugan R Abrha AG. Knowledge and practice of immediate new-born care among midwives in central zone public health facilities, Tigray, Ethiopia: cross sectional study. BMC Res Notes. (2019) 12:487. 10.1186/s13104-019-4532-5 31387618 PMC6685261

[38] Ayenew A Abebe M Ewnetu M. Essential newborn care and associated factors among obstetrical care providers in awi zone health facilities, northwest Amhara Ethiopia: an institutional-based cross-sectional study. Pediatric Health Med Ther. (2020) 11:449–58. 10.2147/PHMT.S276698 33204205 PMC7667187

[39] Esan DT Opeyemi AA Cecilia BB Modupe CO. Knowledge and practices of immediate newborn care among midwives in selected health care facilities in Ekiti state, Nigeria. Pan Afr Med J. (2020) 37:263. 10.11604/pamj.2020.37.263.24628 33598078 PMC7864274

[40] Devi YN Mahadalkar P Varghese R. To assess the knowledge and self reported practices regarding immediate newborn care among the staff nurses from birthing units of selected hospitals of Pune city. Int J Appl Res. (2017) 3(6):499–502. Available online at: https://www.allresearchjournal.com (accessed January 21, 2024).

[41] Sintayehu Y Desalew A Geda B Shiferaw K Tiruye G Mulatu T Knowledge of basic neonatal resuscitation and associated factors among midwives and nurses in public health institutions in eastern Ethiopia. Int J Gen Med. (2020) 13:225–33. 10.2147/IJGM.S255892 32547164 PMC7266389

